# Association between Chronic Pain and Alterations in the Mesolimbic Dopaminergic System

**DOI:** 10.3390/brainsci10100701

**Published:** 2020-10-02

**Authors:** Seoyon Yang, Mathieu Boudier-Revéret, Yoo Jin Choo, Min Cheol Chang

**Affiliations:** 1 Department of Rehabilitation Medicine, Ewha Woman’s University Seoul Hospital, Ewha Woman’s University School of Medicine, Seoul 07804, Korea; seoyonyang@gmail.com; 2 Department of Physical Medicine and Rehabilitation, Centre Hospitalier de l’Université de Montréal, Montreal, QC H2W 1T8, Canada; mathieu.boudier.reveret@gmail.com; 3 Production R&D Division Advanced Interdisciplinary Team, Medical Device Development Center, Daegu-Gyeongbuk Medical Innovation Foundation, Deagu 41061, Korea; cyj361@hanmail.net; 4 Department of Rehabilitation Medicine, College of Medicine, Yeungnam University, Daegu 42415, Korea

**Keywords:** mesolimbic system, dopaminergic system, chronic pain, dopaminergic drug, depression, addiction

## Abstract

Chronic pain (pain lasting for >3 months) decreases patient quality of life and even occupational abilities. It can be controlled by treatment, but often persists even after management. To properly control pain, its underlying mechanisms must be determined. This review outlines the role of the mesolimbic dopaminergic system in chronic pain. The mesolimbic system, a neural circuit, delivers dopamine from the ventral tegmental area to neural structures such as the nucleus accumbens, prefrontal cortex, anterior cingulate cortex, and amygdala. It controls executive, affective, and motivational functions. Chronic pain patients suffer from low dopamine production and delivery in this system. The volumes of structures constituting the mesolimbic system are known to be decreased in such patients. Studies on administration of dopaminergic drugs to control chronic pain, with a focus on increasing low dopamine levels in the mesolimbic system, show that it is effective in patients with Parkinson’s disease, restless legs syndrome, fibromyalgia, dry mouth syndrome, lumbar radicular pain, and chronic back pain. However, very few studies have confirmed these effects, and dopaminergic drugs are not commonly used to treat the various diseases causing chronic pain. Thus, further studies are required to determine the effectiveness of such treatment for chronic pain.

## 1. Introduction

The most current definition of pain, published in May 2020 by the International Association for the Study of Pain (IASP), is “an unpleasant sensory and emotional experience associated with, or resembling that associated with, actual or potential tissue damage” [1]. Pain is different from nociception, which relies on the central nervous system and the peripheral pain signaling pathway. Pain is an integration of nociception with emotion and consciousness. It is always a personal experience and usually plays an adaptive role, although it sometimes has negative consequences on function and psychological wellbeing [1]. Pain that lasts for more than 3 months is defined as chronic pain [2]. Sometimes, chronic pain can be easily treated; however, in many cases, it is extremely difficult to manage and decreases the quality of life, and in severe cases, it may reduce the patient’s occupational abilities [2]. Additionally, patients with chronic pain often develop affective disorders and increased risk of drug addiction [3]. Up to 85% of patients with chronic pain are known to have depression, and half of adults with anxiety or a mood disorder reported experiencing chronic pain [4,5,6]. Moreover, it was reported that drug dependence was presented in 40% of patients with chronic noncancer pain [7].

Chronic pain is sustained in part by central sensitization. In patients with chronic pain, glial cells in the spinal cord and brain release and activate proinflammatory cytokines and chemokines, which cause neuroinflammation and in turn, drive central sensitization [8]. Structural and chemical changes in the brain are also observed in patients with chronic pain, along with altered homeostasis of the dopaminergic system [9,10,11]. Furthermore, several studies have attempted to reduce chronic pain using dopamine-related drugs [12,13,14].

The mesolimbic dopaminergic system is thought to play a primary role in the reward response. It connects the ventral tegmental area (VTA), a major dopamine-producing area in the brain, with several other areas containing dopaminergic receptors [15]. The functional disturbance of this system can cause neuropsychiatric problems, such as depression and drug addiction. Recently, studies have reported that its altered function or anatomy can contribute to the development of chronic pain [3]. Therefore, many clinicians and researchers have focused on the therapeutic potential of manipulating the mesolimbic system to alleviate chronic pain [11,16,17]. Additionally, the use of dopaminergic drugs to compensate for dopamine depletion in the brains of chronic pain patients has been studied, and the results have been discussed [12,13,14].

This review aims to outline the association of the mesolimbic system and the dopaminergic pathway with chronic pain and explore the potential of dopaminergic drugs in controlling chronic pain.

## 2. Dopamine

Dopamine is a central nervous system neurotransmitter with excitatory properties [18]. It belongs to the catecholamine family, which includes dopamine, norepinephrine, and epinephrine. Dopamine is the first catecholamine made in the biosynthetic pathway, produced by the decarboxylation of L-3, 4-dihydroxyphenylalanine (dopa) by aromatic amino acid decarboxylase [18]. Norepinephrine and epinephrine are derived from further metabolic modification of dopamine. As dopamine is a monoamine neurotransmitter, its synthesis is limited by tyrosine hydroxylase [18]. Dopamine is stored in vesicles that are released into the synaptic cleft; this release is controlled by phasic and tonic transmission [19]. At the synapse, dopamine binds to dopamine receptors. There are five different types of dopamine receptors (D1, D2, D3, D4, and D5), each with different pharmacological, biochemical, and physiological functions [20]. These receptors are divided into two receptor families: the D1-like receptor family including D1 and D5 receptors, and the D2-like receptor family including D2, D3, and D4 receptors [21]. Among these, the D2 receptor is the one principally associated with chronic pain. The D2 receptor is expressed in the entire brain, with highest levels of expression in the basal ganglia, globus pallidus, substantia nigra (SN), and VTA [22]. After the postsynaptic neuron elicits an action potential, dopamine is quickly released from the receptor and reabsorbed into the presynaptic cell though the dopamine transporter or the plasma membrane monoamine transporter [23].

Dopamine is responsible for many functions in the brain, including actions and perceptions, voluntary movements, motivation, punishment and reward, inhibition of prolactin production, sleep, mood, attention, working memory, and learning [24,25,26]. Recently, it was also shown to be involved in the control of chronic pain [11]. Previous studies demonstrated a disruption of dopamine homeostasis in the central nervous system in chronic pain patients [27]. Moreover, it was reported that dopaminergic pathways contribute to the progression of subacute pain to chronic pain [28].

## 3. Mesolimbic System

**Figure 1:**
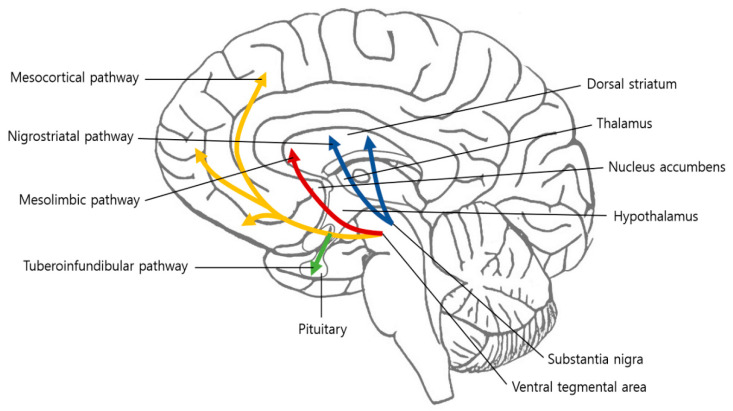
Four major dopaminergic pathways in the brain: the nigrostriatal, mesolimbic, mesocortical, and tuberoinfundibular pathways.

The brain has eight circuits that transmit dopamine, of which the four major pathways are the mesolimbic, mesocortical, nigrostriatal, and tuberoinfundibular pathways (Figure 1) [28]. The remaining four minor pathways are from the VTA to the hippocampus, amygdala, cingulate cortex, and olfactory bulb [28]. The mesolimbic pathway is a central nervous system circuit in which dopaminergic inputs from the VTA innervate brain regions involved in executive, affective, and motivational functions, including the nucleus accumbens (NAc), prefrontal cortex (PFC), anterior cingulate cortex (ACC), and amygdala [11]. Originally, the mesolimbic pathway was only thought to mediate pleasure and goal-directed movement associated with reward-related stimuli [29].

Dysfunction of the mesolimbic system can contribute to neuropsychiatric diseases, including major depressive disorder (MDD) and addiction [30]. In addition, the mesolimbic system plays a major role in the perception and modulation of chronic pain symptoms, and thus this system is considered an important target for pain treatment [11,12,13,14]. The fact that damage to the mesolimbic system is associated with chronic pain, depression, and addiction is likely related to the observation that many patients with chronic pain suffer from depression or drug addiction. Recent studies have demonstrated that the mesolimbic reward circuitry is related to the pathology of chronic pain [31,32]. In an animal study involving mice, it was reported that a peripheral nerve injury causes activation of microglia within mesolimbic system, which results in disruption of dopaminergic signaling and reward behavior [33]. Moreover, disrupted reward circuitry is seen in patients whose chronic pain is rooted in various disorders such as fibromyalgia and burning mouth syndrome [34].

**Figure 2:**
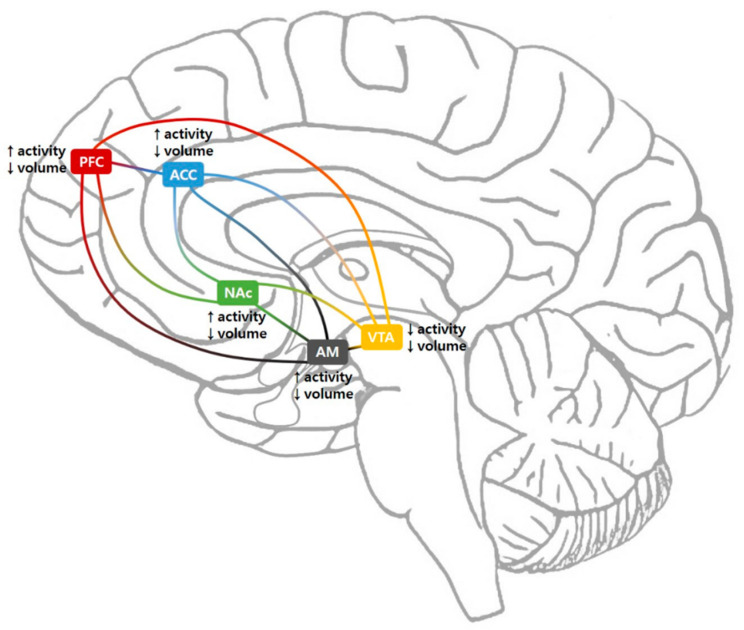
Structural and functional changes within the mesolimbic circuitry in patients with chronic pain. ACC, anterior cingulate cortex; AM, amygdala; NAc, nucleus accumbens; PFC, prefrontal cortex; VTA, ventral tegmental area.

The dopaminergic pathway transmits dopamine from one region of the brain to another (Figure 2). These neurons contain axons that run from dopamine synthesis sites to dopamine target sites [18]. The cell bodies of the neurons are clustered in the VTA and SN of the midbrain [35]. An enzyme capable of forming dopamine and its precursor is made in the cell body of the neuron, which travels along the axons and produces most of the dopamine at the terminal end of the neuron near the synapse; this allows dopamine secretion in the synapse [19]. Studies using positron emission tomography (PET) scans and functional magnetic resonance imaging (fMRI) have reported that pre- and postsynaptic states of dopaminergic neurotransmission are compromised in patients with chronic pain [36].

### 3.1. Ventral Tegmental Area

The VTA is a group of neurons located close to the midline, on the floor of the midbrain. Normal mesolimbic function and affective processes, such as reward-mediated drive, are highly dependent on dopaminergic neurotransmission emanating from the VTA [37,38]. Pain relief is thought to be signaled as a reward via VTA dopaminergic neurons. However, VTA dopaminergic neurons undergo plasticity in patients with chronic pain. Huang et al. damaged the sciatic nerve of mice, finding that action potential firing patterns of VTA dopaminergic neurons were altered [39]. Moreover, when patients with fibromyalgia underwent pain stimulation, the activity of the VTA decreased; in patients with trigeminal neuralgia, the ventral diencephalon volume diminished [40,41]. Akram et al. reported successful treatment outcomes through VTA deep brain stimulation in 21 patients with intractable chronic cluster headaches [42]. About 18 months after initiating deep brain stimulation treatment, they observed a 60% improvement in headache frequency and 30% improvement in headache severity. These outcomes support the role of reduced VTA activity in chronic pain. In addition, disrupted reward function of the VTA appears to be correlated with the development of chronic pain. Loggia et al. performed fMRI in 31 patients with fibromyalgia and compared the results with 14 healthy subjects [34]. They induced pain using a blood pressure cuff inflated at the level of the leg and found that activity in the VTA during periods of anticipated pain relief was reduced in patients with fibromyalgia. The authors suggest that this finding could be correlated with reduced efficacy of medications for pain control in fibromyalgia patients. In addition, connectivity between the VTA and other regions is reduced in patients with chronic pain, depression, and addiction [41].

### 3.2. Nucleus Accumbens

The NAc is a region in the basal forebrain rostral area, located below the corpus striatum [43]. The NAc is connected to the VTA and other structures in the mesolimbic system, such as the amygdala, PFC, and ACC. The NAc receives dopamine and plays an important role in motivation and compensation [44]. Previous fMRI studies have reported that correlated activities between the NAc and other regions in the mesolimbic system are involved in sensory and emotional aspects of pain sensation and its modulation [45,46,47]. This involvement seems to be mediated by signaling reward and motivation. Chronic pain is also reportedly associated with a change of signals between the NAc and other mesolimbic structures [45,46,47]. Selley et al. reported that intraplantar formalin injection in rats produced attenuation of dopaminergic receptor signaling processes in NAc core. They think that this change would be related to the development of a neuropathy-induced allostatic state [48]. In patients with chronic pain, depression, and addiction, the NAc volume decreases and its activity increases [49,50]. Studies have shown that deep brain stimulation of the NAc is effective in treating depression and addiction [51]. Recently, Makary et al. performed fMRI on 40 patients with subacute back pain, 28 patients with chronic back pain, and 30 healthy subjects [52]. They found that the volume of the NAc was significantly reduced in patients with subacute and chronic back pain when compared with that of healthy subjects. In addition, they observed that low-frequency (0.01 to 0.027 Hz) oscillations that occur in the NAc when at rest reduce when back pain progresses from subacute to chronic phase. In addition, connectivity between the NAc and PFC increases in patients with chronic pain. This increase is involved in the transition from acute to chronic pain [53,54].

### 3.3. Prefrontal Cortex

The PFC is the anterior part of the frontal lobe and plays an important role in various cognitive functions such as planning complex cognitive behavior, decision making, memory, and social interactions. The PFC is well-connected to several brain areas, including the limbic system and parietal and temporal lobes [55]. In addition, the PFC is a major component of the mesolimbic system and is involved in pain perception, emotion control, motivation, and substance addiction [56]. In patients with chronic pain, the PFC becomes thin. Seminowicz et al. acquired magnetic resonance imaging (MRI) scans from 16 patients with lower back pain before and 6 months after treatment and compared the results with those of 16 healthy subjects [57]. Patients with lower back pain had reduced cortical thickness in the left dorsolateral PFC compared with healthy subjects. After treatment, the cortical thickness in patients with lower back pain increased. Moreover, the increased thickness of the frontal cortex was associated with improved functional abilities after treatment. Increased activity in the left dorsolateral PFC during an attention-demanding cognitive task observed in the pre-treatment phase became normalized after treatment. In addition, the connectivity of other brain regions to the PFC was found to be changed in patients with chronic pain [57,58]. Studies have revealed that connectivity between the insula and PFC increases in patients with chronic back pain, rheumatoid arthritis, and fibromyalgia [58]. Another study demonstrated pain reduction in patients with fibromyalgia when repetitive transcranial magnetic stimulation (TMS) was applied to the PFC, an observation that may explain the normalization of altered connectivity [59]. Lee et al. applied low-frequency repetitive TMS (rTMS) to the right dorsolateral PFC, high-frequency rTMS to the left dorsolateral PRC, or sham stimulation to 15 women with fibromyalgia [16]. After low-frequency rTMS, depressive symptoms were decreased at 1 month follow-up. Furthermore, high-frequency rTMS immediately and significantly reduced pain and increased quality of life.

### 3.4. Anterior Cingulate Cortex

The ACC is associated with emotion regulation, motivation, evaluation of performance, and identification of errors [60]. As part of the mesolimbic system, it is also involved in the development of chronic pain [61]. In particular, the ACC is associated with the affective or emotional aspects of pain [61]. fMRI studies have shown the recruitment of the ACC in pain processing and found an association between the activation of the ACC and pain-like aversive behavior; inhibition of the ACC prevents such behavior [62]. In patients with chronic pain, the volume of the ACC decreases and the activity of ACC and its connectivity with the periaqueductal gray and insular cortex increases [63]. Several imaging studies have reported increased neuronal activity of the ACC during acute pain stimulation or conditions of persistent pain [64]. Russell et al. compared the volume of gray matter in the ACC between 28 patients with hand osteoarthritis and 11 non-osteoarthritis control subjects by analyzing T1-weighted MRIs of the brain [65]. They found that the gray matter volume in the ACC of patients with hand osteoarthritis was significantly reduced when compared to subjects without osteoarthritis. Alongside ACC volume, that of the insular cortex and thalamus was equally reduced in patients with osteoarthritis. Rainville et al. reported that hypnotic suggestions to decrease pain perception prior to and during noxious stimulation decreased the activity of the ACC and ratings of pain unpleasantness [17]. Studies reveal that bilateral anterior cingulotomy can effectively control chronic pain and MDD symptoms and that application of deep brain stimulation to the ACC can be used as an effective treatment for depression, chronic pain, and cocaine addiction [11,66]. Moreover, serotonin and norepinephrine reuptake inhibitors are known to help in reducing pain in patients with fibromyalgia [67]. Serotonin and norepinephrine reuptake inhibitors can decrease functional connectivity within the ACC-insular cortex-periaqueductal gray network, which appears to contribute to pain reduction [67].

### 3.5. Amygdala

The amygdala is an almond-shaped group of cells in the rostromedial part of the temporal lobe internal to the uncus [68]. The amygdala plays an important role in emotion, memory, and social behavior, and has a role in fear processing [69]. This also involves the emotional affective aspects of pain in the form of regulating emotional stress and pain perception [3,69]. The amygdala receives nociceptive inputs from cortical and thalamic areas, and the lateral/basolateral complex of the amygdala adds affective or emotional context to nociceptive information [3]. Subsequently, this information is sent to the central nucleus of the amygdala, which comprises γ-aminobutyric acid (GABA)-ergic neurons and regulates fear and pain [3]. Gonçalves et al. observed that neuropathic pain due to peripheral nerve injury promotes generation of new neurons in the amygdala. The authors proposed that these neuroplastic changes would contribute to the development of chronic pain-related depressive symptoms [70].

In patients with chronic pain, depression, or addiction, activity of the amygdala is increased and its volume is decreased, as seen in other mesolimbic system structures apart from the VTA [71,72,73]. In cases of chronic pain caused by disorder, such as irritable bowel syndrome, arthritis, and mononeuropathy, activation of the amygdala is increased [74]. Vachon-Presseau et al. performed MRI studies on 159 patients with subacute back pain and 29 healthy subjects [75]. The volume of the amygdala in patients with subacute back pain was smaller than that in normal subjects. In addition, it was reported that small volume of the amygdala is indicative of high risk of transition to chronic pain [52]. Studies have also shown that the use of oral morphine for 1 month in patients with chronic pain decreases gray matter volume in the amygdala and connectivity between the amygdala and ACC [76]. Furthermore, the resting state functional connectivity between the amygdala and the insula, primary motor cortex, primary sensory cortex, and supplementary motor area was found to be increased in patients with irritable bowel syndrome [77]. This connectivity was observed to be reinforced as the intensity of pain increased.

Overall, the volumes of all structures that make up the mesolimbic system appear to be lower in patients with chronic pain. The activity of the VTA is decreased, whereas that of other brain structures constituting the mesolimbic system is increased. This may be in order to compensate for reduced dopamine secretion. The connectivity of the mesolimbic system is decreased in some cases and increased in others.

## 4. Administration of Dopaminergic Drugs

The activity of mesolimbic system-related structures is reduced in patients with chronic pain, and studies have shown that chronic pain is effectively controlled by applying deep brain stimulation to one of the structures in the mesolimbic system [78]. Applying rTMS or transcranial direct current stimulation (tDCS) to the premotor cortex or primary motor cortex has been reported to be effective in reducing chronic pain [79,80,81]. This suggests that stimulation of the brain cortex by rTMS or tDCS may directly or indirectly improve activity of mesolimbic structures and normalize the altered connectivity between affected mesolimbic structures [79,80,81]. There have also been attempts to control chronic pain by administering dopaminergic drugs to supplement decreased dopamine secretion in the mesolimbic system in patients with chronic pain. Studies have attempted to control pain caused by Parkinson’s disease, restless legs syndrome, fibromyalgia, dry mouth syndrome, lumbar radicular pain, and chronic back pain with dopaminergic drugs [12,13,14,82,83,84].

About 45% of patients with Parkinson’s disease experience pain with various clinical features such as aching, numbness, tingling, lancinating, and burning pain, usually in the extremities [82]. This pain could be related to a dysfunction in the central nervous system and dopamine deficit as a manifestation of the disease process [82]. Damage to the basal ganglia and dopaminergic deficit could alter the perception of pain and activity of several areas related to the transfer of nociceptive input, resulting in the development of pain in patients with Parkinson’s disease [82]. Regarding the effect of dopaminergic drugs on pain in patients with Parkinson’s disease, in 2005, Brefel-Courbon et al. observed that the pain threshold of 18 patients with Parkinson’s disease was decreased but was normalized after the administration of dopaminergic drugs (levodopa and/or dopamine agonists) [82]. Moreover, using PET, they investigated changes in cerebral activity after the application of dopaminergic drugs. During the off condition, pain-induced activation was significantly increased in the right insula and PFC and in the left ACC in patients with Parkinson’s disease, as compared to control subjects. These increased activities were normalized by the administration of dopaminergic drugs. In 2007, Gerdelat-Mas et al. also found that the pain threshold in 13 patients with Parkinson’s disease was decreased, and then normalized after administration of 100 mg oral levodopa [83]. In the same year, Slaoui et al. confirmed the same phenomenon (normalized pain threshold using levodopa) in 20 patients with Parkinson’s disease [84].

Restless legs syndrome is a movement disorder characterized by unpleasant sensations and pain in the legs, accompanied by sensory alterations such as hyperalgesia or hyperesthesia [85]. Previous PET studies have revealed abnormalities in the dopaminergic system, such as hypofunction of the mesolimbic and nigrostriatal dopaminergic pathways [85,86]. Treatment with L-Dopa or dopamine agonists significantly reduced unpleasant sensations, pain, and sensory dysfunction in patients with restless legs syndrome [87]. These positive results of the use of dopaminergic drugs support the hypothesis that dopaminergic impairment can contribute to the development of symptoms of restless legs syndrome.

The pain-reducing effects of dopaminergic drugs were also evaluated on other types of chronic pain, such as fibromyalgia, dry mouth syndrome, lumbar radicular pain, and chronic back pain. Fibromyalgia is characterized by chronic widespread pain and bodily tenderness and occurs mainly in middle-aged women [88]. It is often combined with various other symptoms including morning stiffness, chronic fatigue, and affective disturbances. The mechanism of development of fibromyalgia has not been clearly elucidated. However, centrally mediated abnormal sensory processing, including inadequate mesolimbic attenuation of adrenergic arousal and dysfunctions of central inhibitory mechanisms, is known to play an important role [89]. A PET study showed that patients with fibromyalgia have abnormalities of regional cerebral blood flow in several brain regions where dopamine plays an important role in modulating the transfer of pain signals [90,91]. In 2005, Holman et al. recruited 60 patients with fibromyalgia [13]. Of these, 40 patients received 4.5 mg oral pramipexole (a second-generation dopaminergic agonist) at night, and the remaining 20 patients received a placebo. Pramipexole simulates dopamine receptors in the brain, binding selectively to the D2 and D3 receptors. D2 agonists are known to decrease N-methyl-D-aspartate-mediated pain by activating a tyrosine kinase receptor and have been proposed as analgesics [92,93]. The additional specificity of pramipexole to D3 receptors would normalize excessive arousal or inadequate mesolimbic attenuation of adrenergic arousal, which is thought to contribute to pain reduction in fibromyalgia [94]. Fourteen weeks after initiating each medication, the degree of pain as measured using the visual analog scale (VAS) decreased by 36% of initial pain after pramipexole administration, and by 9% of initial pain after placebo administration. Patient function was also significantly improved, and fatigue was significantly reduced.

In 2008, Stuginski-Barbosa reported the successful treatment of refractory dry mouth syndrome with pramipexole (0.125 mg at night) [14]. Burning mouth syndrome is an orofacial neuropathic pain condition affecting the central and peripheral nervous systems. Previous studies showed dopamine hypofunction in the nigrostriatal system in patients with burning mouth syndrome [95,96]. Jääskeläinen et al. showed reduced 6-[^18^F]fluorodopa uptake and increased dopamine D2/D3 receptor availability in the putamen of patients with burning mouth syndrome [95,96]. One week after initiation of treatment with pramipexole, one patient’s pain score, as measured using VAS, decreased from 7 to 5. The dose was slowly increased to 0.75 mg at night. After 4 weeks of treatment, the pain completely disappeared. In 2018, Haddad et al. preformed a randomized controlled crossover study to evaluate the effect of apomorphine (dopamine agonist with high binding affinity to D2, D3, and D5 receptors) on controlling chronic lumbar radicular pain [12]. They recruited 38 patients with chronic lumbar radicular pain and allocated them into two groups: 17 patients in one group and 21 patients in the other. Two hours after subcutaneous injection of 1.5 mg apomorphine, cold pain threshold and tolerance in the hand significantly increased as compared to the baseline. In contrast, after placebo injection, there were no changes in cold pain threshold and tolerance. They concluded that dopaminergic drugs have the potential to control chronic neuropathic pain. They proposed that apomorphine, apart from its central effect, could increase skin blood flow and hence alter the response to the cold pain stimuli.

Chronic back pain is one of most common musculoskeletal disorders causing chronic pain and disability [97]. Clinicians employ several treatment methods such as anti-inflammatory oral medications, epidural steroid injection, pulsed radiofrequency, and physical therapy; in many cases, back pain is refractory to these treatments [98,99,100]. Zeng et al. reported successful treatment with levodopa (125 mg, twice a day) in two patients with chronic lower back pain [101]. The degree of pain in these patients, as measured using VAS, reduced from 6 and 3–5 to 2 and 0, respectively. The patients experienced not only a reduction in lower back pain, but equal relief from hip and shoulder joint pain and pain from restless legs syndrome. We believe that dopaminergic drugs offer a useful option for patients in whom back pain is refractory to conventional treatments.

Aside from dopaminergic drugs, bupropion inhibits dopamine transporters and may exert an analgesic effect by increasing dopamine levels in the NAc [102]. Tramadol increases the expression of D2 and D3 receptors in the NAc, which can contribute to pain reduction [102]. In addition, massage therapy has been reported to increase the levels of dopamine and serotonin while decreasing cortisol levels [103].

Despite the above positive results showing the pain-reducing effect of dopaminergic drugs, it is difficult to draw a clear conclusion due to the small number of existing studies and subjects in each study. However, we find that dopaminergic drugs have some potential to reduce chronic pain and prevent progression to chronic pain. To clarify the effectiveness of dopaminergic drugs, further well-controlled studies should be conducted. Moreover, accurate indications for the use of dopaminergic drugs should be identified by carrying out studies on various diseases that cause chronic pain.

## 5. Conclusions

Decreased dopamine secretion in the mesolimbic system is associated with the development of chronic pain. The mesolimbic system, which is associated with chronic pain, is also closely related to the incidence of depression and addiction. There is a significant neurophysiological overlap amongst chronic pain, depression, and addiction, and thus these conditions are likely to occur simultaneously in a patient [104,105]. By reviewing the existing literature, we conclude that dopaminergic drugs offer an effective treatment for several types of chronic pain. However, the quality of previous studies on the effect of dopaminergic drugs on chronic pain appears to be inadequate. Therefore, in order to prescribe the use of dopaminergic drugs to patients with chronic pain, further well-qualified clinical studies should be conducted to demonstrate the effectiveness of these drugs in treating chronic pain.

## Abbreviations

VTA	ventral tegmental area
NAc	nucleus accumbens
PFC	prefrontal cortex
ACC	anterior cingulate cortex
MDD	major depressive disorder
MRI	magnetic resonance imaging
PET	positron emission tomography
fMRI	functional magnetic resonance imaging
TMS	transcranial magnetic stimulation
rTMS	repetitive transcranial magnetic stimulation
tDCS	transcranial direct current stimulation
VAS	visual analogue scale
